# Characterization of Hormone-Dependent Pathways in Six Human Prostate-Cancer Cell Lines: A Gene-Expression Study

**DOI:** 10.3390/genes11101174

**Published:** 2020-10-07

**Authors:** Andras Franko, Lucia Berti, Alke Guirguis, Jörg Hennenlotter, Robert Wagner, Marcus O. Scharpf, Martin Hrabĕ de Angelis, Katharina Wißmiller, Heiko Lickert, Arnulf Stenzl, Andreas L. Birkenfeld, Andreas Peter, Hans-Ulrich Häring, Stefan Z. Lutz, Martin Heni

**Affiliations:** 1 Department of Internal Medicine IV, Division of Diabetology, Endocrinology, and Nephrology, University Hospital Tübingen, 72076 Tübingen, Germany; andras.franko@med.uni-tuebingen.de (A.F.); robert.wagner@med.uni-tuebingen.de (R.W.); andreas.birkenfeld@med.uni-tuebingen.de (A.L.B.); hans-ulrich.haering@med.uni-tuebingen.de (H.-U.H.); s.lutz@bad-sebastiansweiler.de (S.Z.L.); martin.heni@med.uni-tuebingen.de (M.H.); 2 Institute for Diabetes Research and Metabolic Diseases of the Helmholtz Centre Munich at the University of Tübingen, 72076 Tübingen, Germany; andreas.peter@med.uni-tuebingen.de; 3 German Center for Diabetes Research (DZD), 85764 Neuherberg, Germany; hrabe@helmholtz-muenchen.de (M.H.d.A.); heiko.lickert@helmholtz-muenchen.de (H.L.); 4 Department for Diagnostic Laboratory Medicine, Institute for Clinical Chemistry and Pathobiochemistry, University Hospital Tübingen, 72076 Tübingen, Germany; alke.guirguis@med.uni-tuebingen.de; 5 Department of Urology, University Hospital Tübingen, 72076 Tübingen, Germany; joerg.hennenlotter@med.uni-tuebingen.de (J.H.); arnulf.stenzl@med.uni-tuebingen.de (A.S.); 6 Institute of Pathology, University Hospital Tübingen, 72076 Tübingen, Germany; Marcus.Scharpf@med.uni-tuebingen.de; 7 Institute of Experimental Genetics, Helmholtz Zentrum München, 85764 Neuherberg, Germany; 8 Institute of Diabetes and Regeneration Research, Helmholtz Zentrum München, 85764 Neuherberg, Germany; katharina.wissmiller@helmholtz-muenchen.de; 9 Institute of Stem Cell Research, Helmholtz Zentrum München, 85764 Neuherberg, Germany; 10 Department of Medicine, Technical University of Munich, 81675 München, Germany; 11 Clinic for Geriatric and Orthopedic Rehabilitation Bad Sebastiansweiler, 72116 Mössingen, Germany

**Keywords:** androgen receptor, estrogen receptor, gene expression, insulin receptor, prostate cancer

## Abstract

Prostate cancer (PCa), the most incident cancer in men, is tightly regulated by endocrine signals. A number of different PCa cell lines are commonly used for in vitro experiments, but these are of diverse origin, and have very different cell-proliferation rates and hormone-response capacities. By analyzing the gene-expression pattern of main hormone pathways, we systematically compared six PCa cell lines and parental primary cells. We compared these cell lines (i) with each other and (ii) with PCa tissue samples from 11 patients. We found major differences in the gene-expression levels of androgen, insulin, estrogen, and oxysterol signaling between PCa tissue and cell lines, and between different cell lines. Our systematic characterization gives researchers a solid basis to choose the appropriate PCa cell model for the hormone pathway of interest.

## 1. Introduction

Genetic and metabolic alterations can both drive the development of prostate cancer (PCa) [1,2]. Metabolic alterations can influence autocrine and paracrine signaling pathways, which are crucial in the carcinogenic processes of PCa [3]. A tumor is a complex milieu and, in addition to prostate-cancer cells, it contains fibroblasts, endothelial cells, and mesenchymal stem cells [4]. These various cells communicate with PCa cells via paracrine factors that drive carcinogenesis [4,5]. For instance, paracrine factors secreted by PCa cells were shown to stimulate osteoblasts, which led to excess bone formation [6]. Metabolic alterations, including obesity, metabolic syndrome, insulin resistance, and diabetes, are known to exacerbate PCa [7,8]. These conditions mediate the aggressivity of PCa via endocrine signaling [1]. The major hormone pathways in PCa are androgen, insulin, estrogen, and oxysterol signaling [1,9,10]. In vitro studies use different cell models that are of diverse origin, and have very different cell-proliferation rates and hormone-response capacities [11,12,13]. Although all these cell lines represent valuable tools to study the development of PCa *in vitro*, whether these cell lines are suitable for studying endocrine pathways, which regulate PCa development in humans, is poorly understood. Therefore, it is vital to use a suitable in vitro model that resembles the physiology of endocrine pathways in human PCa. To characterize the major hormone pathways in human PCa cell lines, we systematically compared six commonly applied PCa cell lines and parental primary cells with (i) each other and (ii) PCa tissue samples of patients with PCa by analyzing their gene-expression patterns.

## 2. Materials and Methods

### 2.1. Cell Culture

Human prostate adenocarcinoma cell lines PC3 and LNCaP, isolated from bone and lymph-node metastasis respectively, were purchased from CLS-Cell line services (Eppelheim, Baden-Württemberg, Germany). DU145 cells (isolated from brain metastasis) were obtained from the DSMZ-German collection of microorganisms and cell cultures (Braunschweig, Niedersachsen, Germany). NCI-H660 cells (isolated from lymph-node metastasis) and MDA-PCa-2b (isolated from bone metastasis) were purchased from the ATCC-American type culture collection (Manassas, VA, USA). CWR-R1ca cells (isolated from human xenograft tumors) and human prostate epithelial cells (HPEC) were purchased from Merck (Darmstadt, Hessen, Germany). HPEC cells were used at passages 3 and 4. All cell lines were propagated according to the supplier’s instructions and maintained in a medium supplemented with 100 IU/mL penicillin, 0.1 mg/mL streptomycin, and 2 mM glutamine (Gibco/Thermo Fisher Scientific, Karlsruhe, Baden-Württemberg, Germany) in a 5% CO_2_ humidified atmosphere at 37 °C. LNCaP cells for 14 days of androgen deprivation were cultured in a basal medium (ATCC #CRL-1740, ThermoFisher Scientific #21875034) containing 2 mM L-glutamine and 10% FBS Good (Pan-Biotech P40-37500, Aidenbach, Freistaat Bayern, Germany). The medium used for androgen deprivation (ThermoFisher Scientific #11835030) was supplemented with 10% charcoal-stripped FBS (Sigma-Aldrich, F6765, Munich, Freistaat Bayern, Germany) and 10 nM dihydrotestosterone (Sigma-Aldrich, D-073) for 14 days. Detailed information regarding culture medium and supplements is given in Table 1. Cells were routinely cultivated until 90% confluency before harvesting.

### 2.2. Human Samples

Newly diagnosed PCa patients who had not received treatment before surgery were recruited prior to radical prostatectomy. Tissue sampling was performed by an experienced uropathologist. PCa tissues were immediately snap-frozen in liquid nitrogen and stored at −80 °C. Hematoxylin and eosin staining was performed on paraffinized samples for histological confirmation. Histopathological features were assessed, and pT- and postoperative Gleason scores were determined [14,15]. Informed written consent was obtained from all participants, and the Ethics Committee of the University of Tübingen (575/2018BO2) approved the protocol according to the Declaration of Helsinki.

### 2.3. Real-Time qPCR

Total RNA was isolated using an AllPrep DNA/RNA/miRNA kit (Qiagen, Hilden, Germany) according to the manufacturer’s description, and cDNA was synthesized (Roche, Basel, Switzerland). Real-time PCR was performed with LightCycler 480 Probes Master (Roche) with universal probe library using LightCycler 480 (Roche) [16]. Delta–delta crossing-point (Cp) values were calculated, and data were normalized to ubiquitin c (*UBC*) [8,15]. For real-time PCR analysis, the following primers and probes were applied: *ESR1* 5′-TCCTAACTTGCTCTTGGACAGG and 3′-GTAGCCAGCAGCATGTCG (probe nr 22), *ESRRA* 5′-GTGGGCGGCAGAAGTACA and 3′-TCAACCACCAGCAGATGAGA (probe nr 3), *CYP46A1* 5′-GCTGGACAACTTCGTCACCT and 3′-CATCACTGTGAACGCCAAGT (probe nr 53), *CCND1* 5′-GACCTTCGTTGCCCTCTGT and 3′-GGTTCAGGCCTTGCACTG (probe nr 87), *TP53* 5′-AAGTCTAGAGCCACCGTCCA and 3′-AGTCTGGCTGCCAATCCA (probe nr 3), *GLUT1* 5′-GGTTGTGCCATACTCATGACC and 3′-CAGATAGGACATCCAGGGTAGC (probe nr 67), *GLUT12* 5′-TGCTGCTTTTTCAATTGGTCT and 3′-AGGAAAGATCTCGCTGAGCA (probe nr 37), *IRS1* 5′-GCCTATGCCAGCATCAGTTT and 3′-TTGCTGAGGTCATTTAGGTCTTC (probe nr 71), *IRS2* 5′-TGACTTCTTGTCCCACCACTT and 3′-CATCCTGGTGATAAAGCCAGA (probe nr 49), *FGFR1* 5′-GGCAGCATCAACCACACATA and 3′-TACCCAGGGCCACTGTTTT (probe nr 42), *CH25H* 5′-CCTTCTTCCCGGTCATCTTC and 3′-GATATCCAGGACCACGAAGG (probe nr 9), *CDKN1A* 5′-CCGAAGTCAGTTCCTTGTGG and 3′-CATGGGTTCTGACGGACAT (probe nr 82), *CDKN1B* 5′-GAGAGCCAGGATGTCAGCG and 3′-TTGTTTTGAGTAGAAGAATCGTCGGT (probe CCTTTAATTGGGGCTCCGGCTAACT), *NOS2* 5′-GCTCAAATCTCGGCAGAATC and 3′-GCCATCCTCACAGGAGAGTT (probe nr 42), *SEPP1* 5′-GGAGCTGCCAGAGTAAAGCA and 3′-ACATTGCTGGGGTTGTCAC (probe nr 38). Primer sequences for *AR, CYP27A1, CYP7B1, ESR2, FOLH1, IGF1R, INSRA, INSRB, KLK3, UBC* and *MKI67* are given in [9]. Primer sequences for *HIF1A, RELA* and *BIRC5* are given in [14]. For the human PCa samples, the gene-expression levels of *GLUT1*, *HIF1A*, *BIRC5*, *NOS2*, *RELA* and *MKI67* were previously studied [15].

### 2.4. Western Blot

Total cellular protein was extracted using RIPA buffer (50 mmol/L Tris-HCl pH 7.4, 150 mmol/L NaCl, 1% NP-40, 0.25% Na-deoxycholate, 1 mmol/L phenyl-methyl-sulfonyl-fluoride, 1 mmol/L dithiothreitol) containing a protease and phosphatase inhibitor cocktail (Roche Molecular Biochemicals, Mannheim, Baden-Württemberg, Germany), and cleared by centrifugation. Protein concentration was determined using a BCA protein assay from Bio-Rad (Hercules, CA, USA). The 20 µg protein lysates were separated on a 4–12% Bis-Tris gel (Invitrogen, Carlsbad, CA, USA). After electrophoresis, proteins were transferred using nitrocellulose ministacks and the iBlot dry-blotting system (Invitrogen, Carlsbad, CA, USA). Membranes were blocked for two hours in Odyssey Blocking Buffer (LiCor, Lincoln, NE, USA) and further incubated with antibodies against androgen receptor (AR, ab133273), prostate-specific antigen (PSA, KLK3, ab53774), β-tubulin (ab21057), prostate-specific membrane antigen (PSMA, FOLH1, ab19071) (Abcam, Cambridge, Cambridgeshire, UK), IGF1R β subunit (D23H3, #9750), and insulin receptor β subunit (L55B10, #3020) (Cell Signaling Technologies, Danvers, MA, USA). IRDye^®^ or AlexaFluor^®^ secondary antibodies (LiCor or Abcam, Cambridge, UK) were used, and signals were detected and quantified using the iBright device (Invitrogen, Carlsbad, CA, USA).

## 3. Results

**Figure 1:**
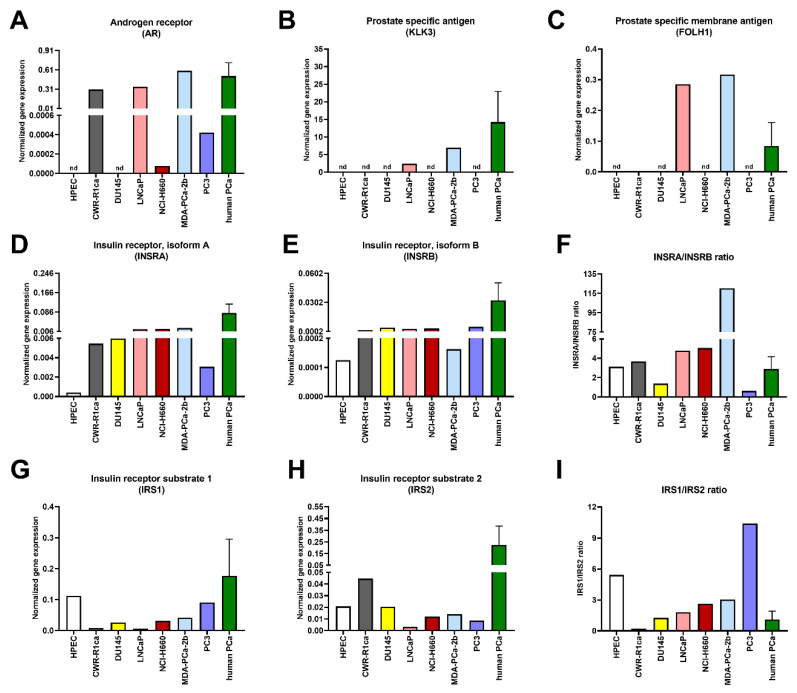
Transcript levels of hormone receptors and downstream substrates in prostate-cancer cell lines and in human prostate-cancer tissue. Transcript levels of indicated genes measured using real-time PCR. (**A**) *AR*, (**B**) *KLK3*, (**C**) *FOLH1*, (**D**) *INSRA*, (**E**) *INSRB*, (**F**) *INSRA/INSRB* ratio, (**G**) *IRS1*, (**H**) *IRS2*, (**I**) *IRS1/IRS2* ratio. PCa: prostate cancer, HPEC: parental primary prostate cells, CWR-R1ca: xenograft PCa cells, DU145: brain metastasis PCa cells, LNCaP: lymph-node metastasis PCa cells, NCI-H660: lymph-node metastasis PCa cells, MDA-PCa-2b: bone metastasis PCa cells, PC3: bone metastasis PCa cells, nd: not detected. For human PCa samples, data shown as pooled samples: mean ± standard deviation (*n* = 11).

**Figure 2:**
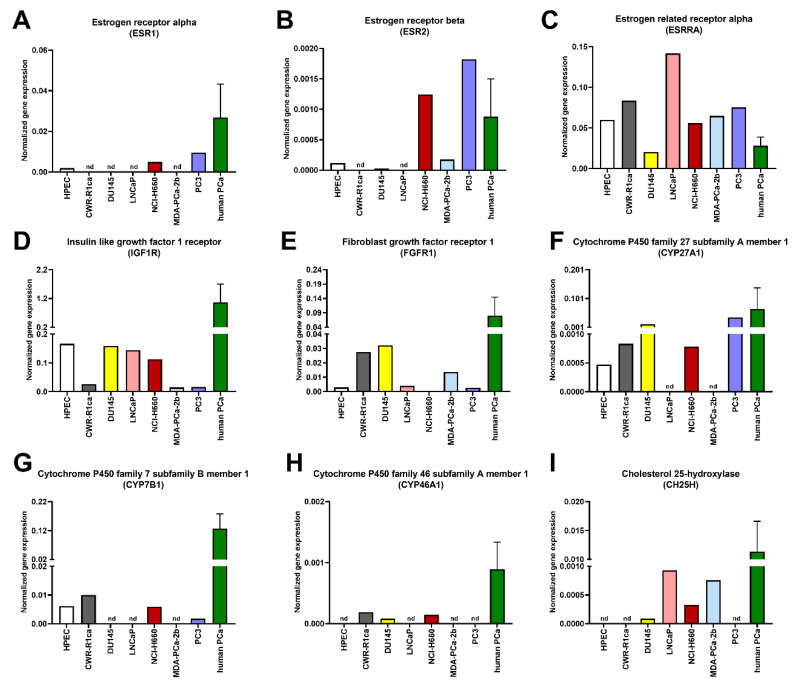
Transcript levels of hormone receptors and potential oncogenic mediators in prostate-cancer cell lines and in human prostate-cancer tissue. Transcript levels of indicated genes were measured using real-time PCR. (**A**) *ESR1*, (**B**) *ESR2*, (**C**) *ESRRA*, (**D**) *IGF1R*, (**E**) *FGFR1*, (**F**) *CYP27A1*, (**G**) *CYP7B1*, (**H**) *CYP46A1*, (**I**) *CH25H*. PCa: prostate cancer, HPEC: parental primary prostate cells, CWR-R1ca: xenograft PCa cells, DU145: brain metastasis PCa cells, LNCaP: lymph-node metastasis PCa cells, NCI-H660: lymph-node metastasis PCa cells, MDA-PCa-2b: bone metastasis PCa cells, PC3: bone metastasis PCa cells, nd: not detected. For human PCa samples, data shown as pooled samples: mean ± standard deviation (*n* = 11).

**Figure 3:**
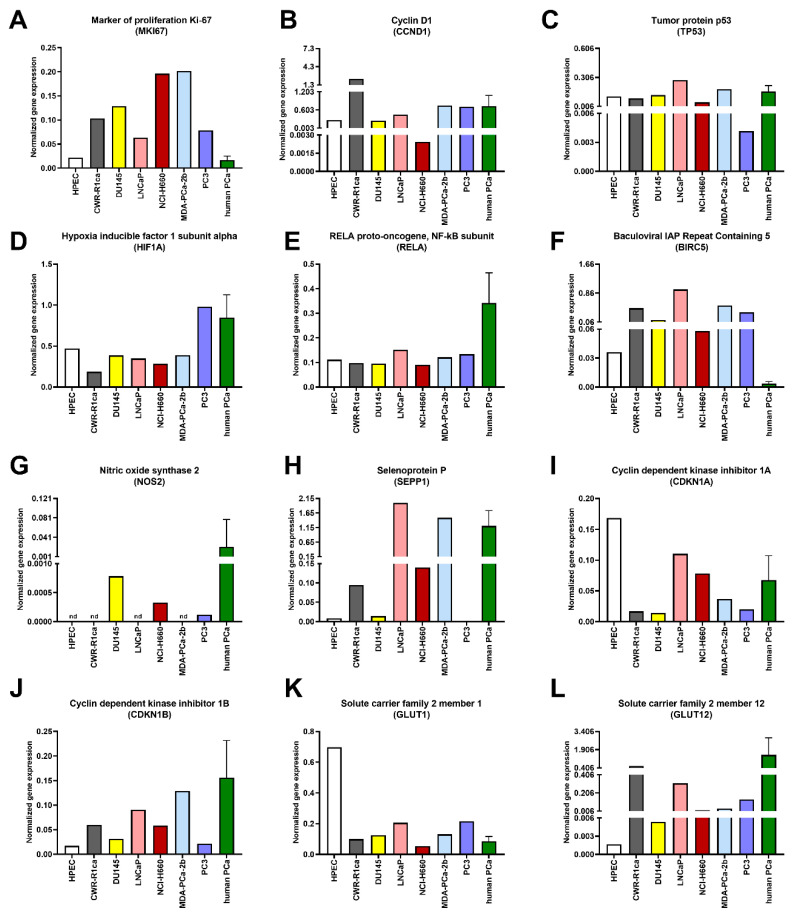
Transcript levels of potential oncogenic mediators in prostate-cancer cell lines and in human prostate-cancer tissue. Transcript levels of indicated genes measured using real-time PCR. (**A**) *MKI67*, (**B**) *CCND1*, (**C**) *TP53*, (**D**) *HIF1A*, (**E**) *RELA*, (**F**) *BIRC5*, (**G**) *NOS2*, (**H**) *SEPP1*, (**I**) *CDKN1A*, (**J**) *CDKN1B*, (**K**) *GLUT1*, (**L**) *GLUT12*. PCa: prostate cancer, HPEC: parental primary prostate cells, CWR-R1ca: xenograft PCa cells, DU145: brain metastasis PCa cells, LNCaP: lymph-node metastasis PCa cells, NCI-H660: lymph-node metastasis PCa cells, MDA-PCa-2b: bone metastasis PCa cells, PC3: bone metastasis PCa cells, nd: not detected. For human PCa samples, data shown as pooled samples: mean ± standard deviation (*n* = 11).

For our comprehensive analysis, we chose six commonly investigated human PCa cell lines (CWR-R1ca, DU145, LNCaP, NCI-H660, MDA-PCa-2b, and PC3). Human prostate epithelial cells (HPEC) were included as parental, nontumorous primary prostate cells. To compare gene expression for hormone pathways in the PCa cell lines to the human situation, we analyzed 11 PCa samples isolated from patients who underwent radical prostatectomy due to their tumor. Histopathological screening confirmed the presence of prostate cancer in the collected tissues. As prostate-cancer metabolism could be different at various tumor stages, we specifically selected patients at a similar tumor stage with comparable Gleason scores (7a and 7b) and without lymph-node metastasis (Table 2). Data for the 11 human samples are shown as pooled values (mean ± standard deviation) in Figure 1, Figure 2 and Figure 3.

As a first step, we quantified the transcripts of the main hormone receptors. The expression levels of androgen receptor (*AR*) and its target genes, prostate-specific antigen (*PSA, KLK3*), and prostate-specific membrane antigen (*PSMA, FOLH1*) were most pronounced in LNCaP and MDA-PCa-2b cells (Figure 1A–C). The insulin receptor isoform A (*INSRA*)/insulin receptor isoform B (*INSRB*) ratio was highest in MDA-PCa-2b cells (Figure 1D–F), suggesting the activation of a mitogenic insulin pathway [17]. PC3 cells demonstrated the largest insulin receptor substrate (IRS) IRS1/IRS2 ratio (Figure 1G–I).

The highest gene levels of estrogen receptors α (*ESR1*) and β (*ESR2*) were observed in NCI-H660 and PC3 cells, whereas the expression of estrogen-related receptor α (*ESRRA*) was the highest in LNCaP cells (Figure 2A–C). The gene-expression levels of insulin-like growth factor 1 receptor (*IGF1R*) were the highest in HPEC and DU145, whereas fibroblast growth factor receptor 1 (*FGFR1*) showed the strongest expression in CWR-R1ca and DU145 cells (Figure 2D–E).

As a second step, we quantified the mRNA levels of further major intracellular regulators of PCa. Among the three investigated enzymes of oxysterol metabolism, the strongest expression for cytochrome P450 family 27 subfamily A member 1 (*CYP27A1*) was observed in DU145 and PC3 cells (Figure 2F), whereas cytochrome P450 family 7 subfamily B member 1 (*CYP7B1*) was highly represented in CWR-R1ca cells (Figure 2G). The transcript for cytochrome P450 family 46 subfamily A member 1 (*CYP46A1*) was detected only in CWR-R1ca, DU145, and NCI-H660 cells (Figure 2H). In order to use a statistical test among the analyzed cells lines, we examined cell-type-dependent gene expressions in a mixed model by bundling different genes to pathways: androgen pathway: *AR*, *PSA*, and *PSMA*; insulin pathway: *INSRA, INSRB, IRS1*, and *IRS2*; estrogen pathway: *ESR1, ESR2,* and *ESRRA*; oxysterols: *CYP7B1, CYP46A1*, and *CYP27A1*. The expressed genes were considered as random effects, and the pathways, cell types, and their interactions as fixed effects. We observed a significant interaction between cell type MDA-PCa-2b and the androgen pathway, which suggested that the MDA-PCa-2b cell line has a different gene expression for this pathway. Cholesterol 25-hydroxylase (*CH25H*) was only identified in four cell lines (Figure 2I).

Next, we analyzed three genes that are involved in proliferative pathways [18,19]. The expression of Ki-67 (*MKI67*) was the highest in NCI-H660 and MDA-PCa-2b cells (Figure 3A). CWR-R1ca cells showed the strongest expression of cyclin D1 (*CCND1*) (Figure 3B). The mRNA level of tumor protein p53 (*TP53*) was high in LNCaP and MDA-PCa-2b cells (Figure 3C). Hypoxia inducible factor 1 subunit α (*HIF1A*) and RELA proto-oncogene NF-kB subunit (*RELA*), as well as the NF-kB target genes, including baculoviral IAP repeat containing 5 (*BIRC5*), nitric oxide synthase 2 (*NOS2*), and selenoprotein P (*SEPP1*), are important regulators of carcinogenic processes in the development of PCa [20,21,22,23]. *HIF1A* was strongly expressed in PC3 cells (Figure 3D). mRNA levels of *RELA* and *BIRC5* were comparable among the cell lines (Figure 3E,F). *NOS2* was detected in three cell lines (Figure 3G). The transcript levels of *SEPP1* were the largest in LNCaP and MDA-PCa-2b cells (Figure 3H). Cyclin-dependent kinases and their inhibitors are implicated in mitogenic signaling and were demonstrated to be regulated by androgen signaling [18]. Cyclin-dependent kinase inhibitor 1A (*CDKN1A*) showed strong gene expression in HPEC cells (Figure 3I). The mRNA levels of *CDKN1B* were the highest in LNCaP and MDA-PCa-2b cells (Figure 3J). Glucose transporters regulate crucial tumorigenic pathways, which have particular interest in the term of diabetes [24]. Among the analyzed glucose transporters, solute carrier family 2 member 1 (*GLUT1*) showed the strongest expression in HPEC cells (Figure 3K). The strongest gene expression for solute carrier family 2 member 12 (*GLUT12*) was found in CWR-R1ca cells (Figure 3L).

Ghandi and colleagues recently assessed the transcript pattern of five PCa cell lines using RNA sequencing [25]. The observed differences of gene-expression pattern among the analyzed cells lines in our study resemble these previous data for DU145, LNCaP, NCI-H660, MDA-PCa-2b, and PC3 cells (Table 3, https://www.cbioportal.org/, and [25]).

**Figure 4:**
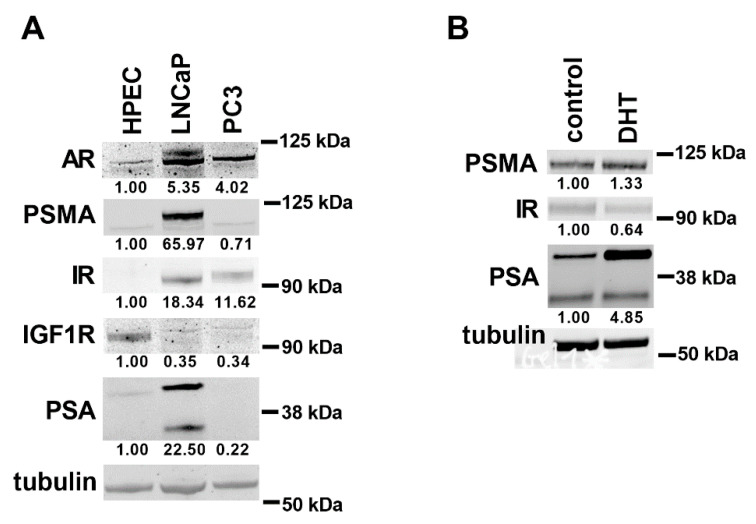
Protein levels of androgen receptor (AR), PSA, prostate-specific membrane antigen (PSMA), insulin receptor (IR), and insulin-like growth factor 1 receptor (IGF1R) in prostate-cancer cell lines. Proteins detected using SDS-PAGE and Western blot. (**A**) For HPEC, LNCaP, and PC3 cell lysates, AR, PSA, PSMA, IR, and IGF1R antibodies were applied. (**B**) For LNCaP cell lysates, PSA, PSMA, and IR antibodies were applied. As loading control, β-tubulin was used. Protein intensities were normalized to (**A)** β-tubulin and HPEC cells or (**B**) control condition. These relative protein intensities are under the pictures. For quantifying PSA intensity, upper protein bands were evaluated. Control: LNCaP cells grown in growth medium; DHT: dihydrotestosterone treatment of LNCaP cells after serum deprivation. Numbers on the right side represent molecular-weight markers.

Furthermore, we measured the protein levels for AR, PSMA, IR, IGF1R, and PSA using Western blotting (Figure 4A). Most of the observed changes at the gene-expression level among HPEC, LNCaP, and PC3 cells were mirrored well on the protein level. The gene-expression profiles of CWR-R1ca, DU145, NCI-H660, and MDA-PCa-2b cells are in line with protein-expression data of previous studies [11,12,26,27,28,29]. As LNCaP cells showed profound PSA and PSMA expressions at the protein level, we treated these cells with dihydrotestosterone (DHT) after serum deprivation. DHT treatment increased the protein level of PSA (Figure 4B), suggesting that LNCaP cells are partly sensitive for hormone treatment after serum deprivation.

## 4. Discussion

All analyzed PCa cell lines had lower insulin-receptor expression levels than those of PCa samples obtained from PCa patients. On the other hand, transcript levels of potential oncogenic mediators and proliferation markers in different prostate-cancer cell lines and in human prostate-cancer tissue have similar expression. These data indicate that certain PCa cell lines can serve as an appropriate model for investigating the main hormone pathways on the transcript level.

Our comprehensive data regarding the mRNA levels of endocrine-signaling components identified major differences among the six human PCa cell lines and the parental nontumorous primary cells. Androgen-receptor signaling is the best-studied pathway in PCa [1,22]. LNCaP and MDA-PCa-2b cells have very high transcript levels for androgen-signaling components; the present data suggest that, out of the six analyzed PCa cell lines, LNCaP and MDA-PCa-2b cells are probably the best choice for studies on androgen signaling (Table 4).

In contrast to the androgen-signaling pathway, there is less knowledge about insulin, estrogen, and oxysterol signaling in PCa cell models. A high insulin-receptor isoform A to insulin-receptor isoform B ratio in PCa suggests the prevailing activation of the mitogenic insulin pathway [17,30]. Our analysis indicates that MDA-PCa-2b cells could be highly responsive to mitogenic insulin signaling (Table 4). On the other hand, NCI-H660 and PC3 cells appear to be suitable for analyzing estrogen signaling due to the high expression levels of *ESR1* and *ESR2* (Table 4). Cholesterol derivates, including oxysterols [31], were recently implicated in the regulation of PCa growth by modulating androgen-receptor signaling [9]. Our current data indicate that, on the mRNA level, the major enzymes of oxysterol metabolism show significant differences in the analyzed PCa cell lines.

Furthermore, the activation of HIF1A and NF-kB pathways plays a pivotal role in oncogenic processes [24,32]. In human prostate samples, we observed a positive correlation of the transcript levels of HIF1A and NF-kB pathways to the oncometabolite fumarate [14]. These real-time PCR data indicate that, for investigating HIF1A pathways *in vitro*, PC3 cells could serve as an appropriate model (Table 4). Patients with Type 2 diabetes develop more aggressive PCa [33,34] and show increased risk of PCa mortality [35]; therefore, altered glucose metabolism and the upregulation of glucose transporters could be involved in the progression of PCa in patients with diabetes [7,24]. HPEC cells had high *GLUT1* expression levels, whereas CWR-R1ca cells showed the highest expression for *GLUT12* transcripts, indicating that these cell lines could be used to analyze the involvement of glucose metabolism in cancer progression.

Androgen-deprivation therapy (ADT) is one of the common treatment options for localized PCa [36] and was shown to modify intracellular hormonal metabolism [37]. PCa cell lines represent in vitro models studying the consequences of ADT and hormone stimulus [13]. In LNCaP cells, PSA showed high androgen sensitivity, which is in line with previous studies [38,39]. Nevertheless, ADT has additional effects on PCa cells, as it also stimulates neuroendocrine differentiation in LNCaP cells [40]; thus, the in vitro application of hormone treatment after serum deprivation should be carefully interpreted.

The cell lines in our study were cultivated in various culture conditions, such as different media, hormonal supplements, and FBS concentrations, following suggestions for each cell line from the provider. Nevertheless, these different conditions could have created a bias on the gene expressions that we could not control.

In summary, our systematic characterization gives researchers a solid basis to choose the most appropriate PCa cell-culture model to characterize the hormone pathway of interest (Table 4).

## Figures and Tables

**Table 1:** Characteristics of the cell lines used in the study. If not otherwise specified, fetal bovine serum (FBS) was purchased from Bio&SELL (FBS Superior, #S 0615, Feucht, Germany).

Cell Line	Origin	Catalog Nr	Medium	Supplements (Final Concentration)
**PC3**	Adult, male Caucasian. Prostate, from metastatic site: bone. Adenocarcinoma, Grade IV	CLS, cell line service #300312	ThermoFisher Scientific #11039047	2 mM L-glutamine, 5% FBS
**LNCaP**	Adult, male Caucasian. Prostate, from metastatic site: left supraclavicular lymph node. Carcinoma	CLS, cell line service #300265	ThermoFisher Scientific #51200046	2 mM L-glutamine, 10% FBS, 0.1 mM nonessential amino acids, 1 mM sodium pyruvate
**MDA-PCa-2b**	Adult, male African American. Prostate, from metastatic site: bone	ATCC #CRL-2422	ATCC #30-2004	20% FBS, 25 ng/mL cholera toxin, 10 ng/mL mouse EGF, 0.005 mM phosphoethanolamine, 100 pg/mL hydrocortisone, 45 nM sodium selenite, 0.005 mg/mL human recombinant insulin
**CWR-R1ca**	Fibroblast-free cell line derived from the castration-resistant or recurrent CWR-R1 human prostate-cancer cell line	Merck-Millipore #SCC118	ThermoFisher Scientific #32404014	2 mM L-glutamine, 10% FBS
**NCI-H660**	Adult, male Caucasian. Prostate, from metastatic site: lymph node	ATCC #CRL-5813	ThermoFisher Scientific #32404014	4 mM L-glutamine, 5% FBS, 0.005 mg/mL insulin, 0.01 mg/mL transferrin, 30 nM sodium selenite, 10 nM hydrocortisone, 10 nM β-estradiol
**DU145**	Adult, male Caucasian. Prostate, from metastatic site: brain	DSMZ #ACC 261	ThermoFisher Scientific #32404014	2 mM L-glutamine, 10% FBS
**HPEC**	Adult, normal	Merck-Millipore #SCCE019	Merck-Millipore #SCMP001 (Kit)	6 mM L-glutamine, 0.4% epiFactor P, 1 µM epinephrine, 0.5 ng/mL rh TGF-α, 100 ng/mL hydrocortisone hemisuccinate, 5 µg/mL human recombinant insulin, 5 µg/mL apo-transferrin

**Table 2:** Patient characteristics. Abbreviations: BMI: body-mass index, PSA: prostate-specific antigen, N: number of patients, Stdev: standard deviation. pT and Gleason scores represent prostate-cancer (PCa) pathological stages; pN denotes lymph-node status.

Patient Characteristics	Mean	Stdev	N
**Age (y)**	65	8	11
**BMI (kg/m^2^)**	26.5	3.2	11
**PSA (ng/mL)**	12.1	8.1	10
**pT–2c**			6
**pT–3a**			4
**pT–3b**			1
**pN**			0
**GLEASON 7a**			7
**GLEASON 7b**			4

**Table 3:** RNA sequencing data from https://www.cbioportal.org/ [25].

Cell Line	DU145	LNCaP	NCI-H660	MDA-PCa-2b	PC3
Gene ID					
***AR***	0.0934	52.35	0.0104	24.77	0.0098
***KLK3***	0.0152	3066.78	0	3292.29	0
***FOLH1***	0.0447	473.17	0.0119	308.79	0.1089
*INSR* *	1.27	5.68	6.41	5.39	1.87
***IRS1***	3.59	2.50	2.15	5.40	7.05
***IRS2***	3.44	0.8080	2.80	6.19	0.7902
***ESR1***	0.0184	0.0015	0.9132	0.0016	0.2013
***ESR2***	0.1613	0.0867	0.5153	0.0632	0.2337
***ESRRA***	11.19	27.04	12.65	29.41	19.22
***IGF1R***	5.41	9.46	6.21	22.71	1.58
***FGFR1***	13.30	2.68	0.3394	3.32	5.23
***CYP27A1***	3.42	0.0650	0.1743	0.0264	5.67
***CYP7B1***	0.0550	0	0.3231	0.0092	0.7905
***CYP46A1***	0.0446	0.2518	0.1962	0.1349	0.1359
***CH25H***	0.0455	0.0151	0.0282	0	0.0157
***MKI67***	58.09	16.15	38.52	26.22	24.39
***CCND1***	129.71	110.32	0.6017	98.12	232.60
***TP53***	32.21	39.17	5.85	17.03	1.01
***HIF1A***	111.95	40.72	54.63	123.32	83.55
***RELA***	27.04	19.18	20.18	19.53	16.76
***BIRC5***	6.84	2.61	4.12	2.57	6.09
***NOS2***	0.5080	0	0.0462	0	0.0081
***SEPP1***	0.6737	27.18	34.10	44.55	1.32
***CDKN1A***	18.73	92.39	52.25	40.94	4.49
***CDKN1B***	17.72	28.15	40.70	32.49	8.18
***GLUT1***	68.77	19.17	10.53	25.15	63.88
***GLUT12***	0.5633	26.02	0.9042	6.98	0.6577

Numbers denote median values.* Applied method did not distinguish between INSRA and INSRB isoforms, which were, however, differentiated by our qPCR analysis.

**Table 4:** Summary table.

Pathway of Interest	Proposed Useful PCa Cell Lines
Androgen signaling	LNCaP, MDA-PCa-2b
Mitogenic insulin signaling	MDA-PCa-2b
Estrogen signaling	NCI-H660, PC3
HIF1A pathway	PC3
